# Morphological Differences in Feeding and Digestive Organs, the Diversity of Intestinal Microorganisms, and Variations in Digestive Enzyme Activity Promote the Differentiation of Nutritional Niches in Schizothoracinae Species

**DOI:** 10.3390/ani15223242

**Published:** 2025-11-08

**Authors:** Taiming Yan, Fei Liu, Mengna Chang, Ruizhen Yan, Wenjie Luo, Lin Wen, Wenxiang Ding, Qipeng Fu, Xuanyu Wang, Xin Li, Hao Song, Kuo Gao, Xiang Wang, Congyu Xu, Rukui Zeng, Ziting Tang, Zhi He, Deying Yang

**Affiliations:** 1 College of Animal Science and Technology, Sichuan Agricultural University, 211 Huimin Road, Chengdu 611130, China; yantaiming@sicau.edu.cn (T.Y.); dingwenxiang@stu.sicau.edu.cn (W.D.); fuqipeng123@gmail.com (Q.F.); wxy171102@126.com (X.W.); 18089561072@163.com (X.L.); 18528538904@163.com (H.S.); 17758707139@163.com (X.W.); xcygood@163.com (C.X.); tangziting@sicau.edu.cn (Z.T.); 2 Yalong River Hydropower Development Company, Ltd., Chengdu 610051, China; zengrukui@ylhdc.com.cn

**Keywords:** cyprinidae, highland river, interspecies competition, adaptation, feeding strategies

## Abstract

**Simple Summary:**

The analysis of feeding strategies in different nutritional niches among five Schizothoracinae species presented that the morphological differences in feeding/digestive organ morphology, diet composition, intestinal digestive enzyme activity, and microbial diversity drove the dietary divergence. The omnivore-fish, *Ptychobarbus leptosomus,* has the higher lipase activity, and exhibits significantly different intestinal microbial composition/diversity compared to the other four fish species. The results of this study can deepen our understanding of the coexistence of Schizothoracinae fishes in high-elevation river ecosystems.

**Abstract:**

The mechanisms of nutritional niche differentiation among sympatric Schizothoracinae fishes play an important role in their adaptive evolution and should be considered in conservation strategies. To date, there have been no reports about the role of different feeding strategies in nutritional niche differentiation among five Schizothoracinae species (*Ptychobarbus leptosomus*, PL; *Gymnodiptychus pachycheilus*, GP; *Schizothorax kozlovi*, SK; *Schizopygopsis malacanthus*, SM; and *S. wangchiachii*, SW). This study explored the role of feeding strategy differences in nutritional niche differentiation among sympatric Schizothoracinae fishes. We analyzed feeding/digestive organ morphology, diet composition, intestinal digestive enzyme activity, and microbial diversity in five species from the middle Yalong River. The results revealed dietary divergence: PL consumed small fish, invertebrates, and algae; GP/SK fed on invertebrates and algae; and SM/SW primarily ate algae. Additionally, α-amylase activity was lower in PL/GP/SK than in SM/SW (*p* < 0.05), while lipase activity was lower in SM/SW than in PL/GP/SK (*p* < 0.05), and PL exhibited the highest trypsin activity (*p* < 0.05). The intestinal microbial composition/diversity also varied: the PL group differed significantly from the GP + SK and SM + SW groups (*p* < 0.05), with *Cetobacterium* being dominant in the PL group and *Clostridium* being dominant in the other groups; the α diversity was highest in the SM + SW group and lowest in the PL group. PICRUSt2 predictions revealed significant differences in nutrient metabolism pathways between the PL group and the other groups (*p* < 0.05). Overall, the morphological differences in feeding/digestive organs and recent food intake may drive the dietary divergence, inducing adaptive changes in digestive enzymes and microbial diversity and ultimately promoting nutritional niche differentiation among sympatric Schizothoracinae species.

## 1. Introduction

Niche theory states that the coexistence of sympatric species depends on niche differentiation [1]. Niche differentiation encompasses differentiated strategies across dimensions such as spatial, temporal, resource, and physiological aspects, including habitat, temporal, and physiological differentiation [2,3]. Nutritional niche differentiation, a subset of resource utilization differentiation, refers to species-specific strategies for food exploitation, nutrient absorption, or metabolic pathways, which reduces competition under resource limitation. This process is widespread among sympatric fish species, facilitating coexistence by optimizing resource allocation [4,5]. Nutritional niche differentiation is frequently associated with morphological or physiological adaptations. Specifically, the structural characteristics of feeding organs—including the mouth, teeth, and gill rakers—exhibit functional correlations with feeding behaviours [6]. For example, several characters (including lower jaw morphology, shape of pharyngeal teeth, the number of pharyngeal teeth rows, and mouth position) of Schizothoracinae from the Qinghai-Tibetan Plateau were either functionally correlated during the evolutionary process or linked to the specific food resource use [7]. These morphological disparities directly modulate food resource utilization and foraging strategies, thereby establishing a fundamental basis for niche partitioning among sympatric fish species [8].

Digestive enzyme activity reflects species-specific digestive physiology, with significant variations across feeding habits [9]. Dietary habits of fish are adapted to the activity of digestive enzymes and the morphology of intestinal tissues in fish. Analysis of digestive enzyme activity and intestinal tissue morphology of fish is helpful to define fish dietary habits, and improve feed formulas and breeding methods [10]. Furthermore, the intestinal microbiota plays a pivotal role in nutrient absorption and niche differentiation via functional specialization. Herbivorous fish depend on cellulose-degrading bacteria such as *Clostridium* and elevated cellulase and amylase activities to digest plant fibres [11]; carnivorous fish employ protease-producing bacteria such as *Cetobacterium* and increased trypsin activity to improve protein absorption; and omnivorous fish harbour a combination of these microbial groups to adapt to diverse dietary regimens [12]. Therefore, the intestinal digestive enzyme activity and intestinal microbial diversity of fish adapt to changes in their dietary habits. These two factors play crucial roles in nutrient metabolism and absorption and collectively promote the nutritional niche differentiation of sympatric fish species.

The Yalong River, a typical highland river in the upper Yangtze Basin, harbours rich ichthyofaunal diversity attributed to its complex hydrological conditions, such as alternating rapids, slow-moving currents, and pools [13]. Schizothoracinae fishes, a dominant taxonomic group in this region, represent ideal biological models for investigating highland adaptation and biodiversity patterns [14]. Over evolutionary timescales, these fishes have evolved morphological characteristics—including keratinized mandibles, pharyngeal teeth structure, and intestinal length—adapted to their feeding habits, enabling their classification into primitive groups such as *Schizothorax kozlovi* (SK) and *S. wangchiachii* (SW), specialized groups including *Ptychobarbus leptosomus* (PL) and *Gymnodiptychus pachycheilus* (GP), and highly specialized groups such as *Schizopygopsis malacanthus* (SM) [15,16,17]. Previous study has demonstrated that SW had the highest alpha diversity indices of gut microbiota (e.g., Chao 1 index) and the highest species dispersal compared to SK in the lower reaches of the Yalong River [12]. Then, GP is an euryphagous and omnivorous species, and had the low overall feeding intensity during the reproduction season in the middle reaches of the Yalong River [18]. The gut microbiota of three wild populations (Longbaotan National Nature Reserve, the upstream and downstream of Batang River) of SM in the Tongtian River system of Yushu Prefecture, Qinghai Province show significant clustering according to different river systems [19]. The above five species coexist in the middle reaches of the Yalong River. Understanding the mechanisms of nutritional niche differentiation among sympatric Schizothoracinae fishes is crucial for elucidating their adaptive evolution and informing conservation strategies. To date, there have been no reports on the differences in feeding strategy as they relate to nutritional niche differentiation among SK, SW, PL, GP, and SM.

In this study, the feeding and digestive organ morphology, diet composition, intestinal enzyme activity, and microbial diversity of the five species were analyzed and compared using morphometric analysis, gut content analysis, spectrophotometry, and high-throughput sequencing, respectively. Our findings provide the foundational insights into nutritional niche differentiation and support conservation efforts for Schizothoracinae populations.

## 2. Materials and Methods

### 2.1. Sample and Trait Data Collection

**Figure 1:**
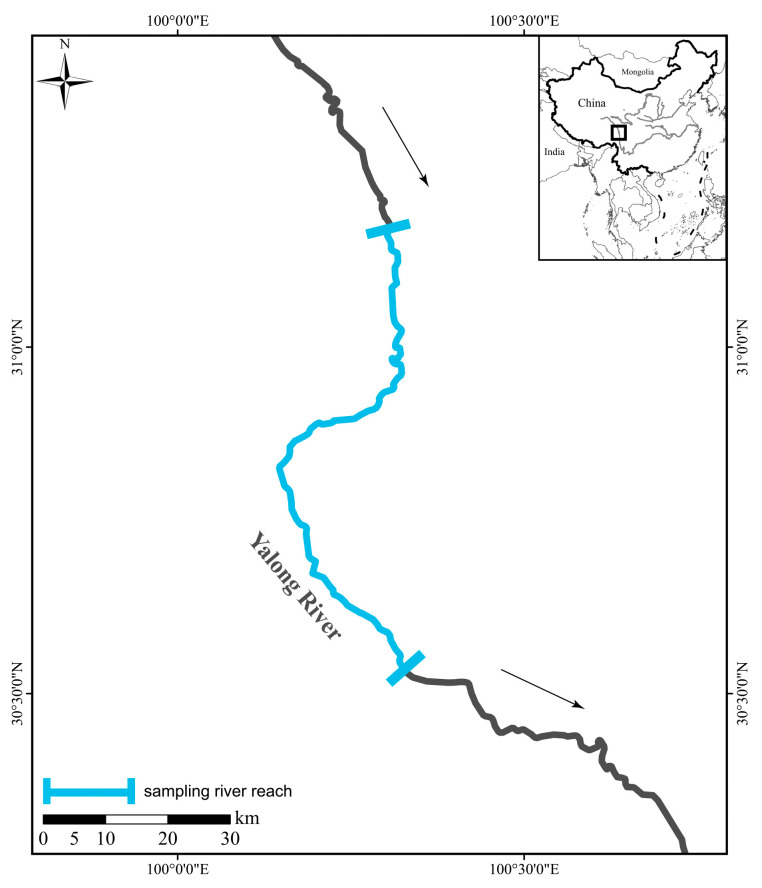
Sampling sites of the five Schizothoracinae species. The arrows indicate the direction of river flow.

Samples of SK, SW, PL, GP, and SM were collected from the Bome Township section (71 km in length) of the middle Yalong River in Xinlong County in May–June 2023 and May–June 2024 (Figure 1). With approval from relevant authorities, sampling was primarily conducted using fixed gillnets, drift gillnets, and ground cages. Morphological identification was performed on the basis of the taxonomic characteristics described in previous studies [20,21]. A total of 25 SM, 29 GP, 27 PL, 13 SW, and 10 SK individuals were obtained. All the experimental procedures involving animal research were approved in January 2023, and were carried out in accordance with the guidelines of the Ethical Committee of Sichuan Agricultural University (Approval Number: 20230131).

The fish were anesthetized with 0.8 g/L MS-222 (Sigma-Aldrich, St. Louis, MO, USA) before standard biological parameters, including body length (to the nearest 0.1 cm) and body weight (to the nearest 0.1 g), were measured. The surface of each fish and the dissection tools were disinfected with 75% ethanol. Intestinal fullness was observed and recorded during dissection, and was graded on a 6-point scale (0–5) using Arabic numerals, where 0 indicated an empty intestine and 5 indicated that the intestine was completely filled with food. For samples with a fullness grade >2, 1 g of intestinal content was collected into 2 mL cryotubes and frozen in liquid nitrogen for high-throughput sequencing analysis of gut microbial diversity. The anterior intestine of each species was obtained, rinsed with normal saline, and freed of adipose tissue. A 1 cm segment of the intestine was then excised, placed into a 5 mL cryotube, and frozen in liquid nitrogen for subsequent determination of digestive enzyme activity.

### 2.2. Observation of Major Feeding Organs and Intestinal Morphology

The direction of the mouth cleft was observed using visual inspection and categorized according to the criteria [22]. The morphological characteristics of the anterior mandible were recorded. After the branchial cavity was dissected, the hypopharyngeal bone was removed and observed under a stereomicroscope (Guangzhou Micro-shot Optoelectronics Technology, Guangzhou, China) at 20× magnification. On each side, the number of rows of pharyngeal teeth and the total number of pharyngeal teeth were recorded from medial to lateral. The first gill arch on the left side was excised, and the number of lateral gill rakers was counted under a dissecting microscope. The coiling pattern of the intestine was observed in situ after dissection, photographed from the ventral side, and then schematically drawn in Adobe Illustrator (2020, v 24.3) [23]. After dissecting and removing the fish intestine, the intestine was naturally straightened, and the length from the anterior end of the intestine to the anal end was measured using a straightedge.

### 2.3. Analysis of Food Composition

The intestinal contents were fixed with 10% formalin and filtered through a 0.5 mm mesh sieve. The entire filtrate was collected and diluted with distilled water to a final volume of 30 mL. Larger food items, such as aquatic insects and fish, in the filter residue were identified and counted using either the naked eye or a stereomicroscope. Smaller food items in the filtrate, including algae, were identified and quantitatively analyzed under an Olympus CX21 microscope [24]. Food types were identified to the lowest possible taxonomic level using references such as *Freshwater Algae of China* (Science Press, Beijing, China) and the *Atlas of Common Freshwater Planktonic Algae in China* (Shanghai Scientific & Technical Publishers, Shanghai, China) [25,26]. Aquatic insects were generally identified to the order level, with some classified to the family or genus level; molluscs, shrimp, and crabs were generally identified to the genus level; and algae were typically identified to the genus level. The detailed methods for the quantitative analysis of small food items followed established protocols [27,28].

### 2.4. Determination of Intestinal Digestive Enzyme Activity

For each species, the similar body length, body weight, and sex in five individuals were selected for the determination of intestinal digestive enzyme activity [29]. Digestive enzyme activities were measured using kits produced by Nanjing Jiancheng Bioengineering Institute (Room 1106, Xinliji Building, No. 258-27 Zhongyang Road, Nanjing, China), including an α-amylase assay kit (C016-1-1, Nanjing, China), a lipase assay kit (A054-1-1, Nanjing, China), and a trypsin assay kit (A080-2-2, Nanjing, China).

### 2.5. Extraction of Gut Microbial DNA and Sequencing

The exaction of total genomic DNA from the microbial community and PCR amplification was followed by a previous study [30]. The barcode-tagged primers of V3–V4 hypervariable region of the *16S rRNA* gene were the forward primer (5′-TACGGRAGGCAGCAG-3′) and reverse primer (5′-AGGGTATCTAATCCT-3′). The PCR products were quantified by a Qubit 4.0 (Thermo Fisher Scientific, Waltham, Massachusetts, USA). The libraries for purified PCR products were constructed by NEXTFLEX Rapid DNA-Seq Kit (Waltham, Massachusetts, USA), and then were sequenced by the Illumina NextSeq 2000 platform (Shanghai Majorbio Biopharm Technology Co., Ltd., Shanghai, China). There are 15 biological replicates for the PL samples, seven for the GP, six for the SK, 20 for the SM, and 13 for the SW. The raw sequencing data were submitted to the NCBI SRA database (accession number: PRJNA1283483).

### 2.6. High-Throughput Sequencing Data Analysis

The raw paired-end sequencing reads of gut microbiota were quality controlled by fastp software (v 0.19.6) and merged using FLASH software (v 1.2.11). Quality-controlled and merged sequences were denoised using the DADA2 (v 1.30.0) plugin in the QIIME2 (v 2024.2) [31] pipeline with default parameters. Sequences were commonly referred to as amplicon sequence variants (ASVs). Chloroplast and mitochondrial sequences annotated were removed. To minimize the impact of sequencing depth on subsequent alpha and beta diversity analyses, the number of sequences per sample was rarefied to 20,000, and the average Good’s coverage per sample remained at 99.09%. Taxonomic classification of ASVs was performed using the naive Bayes classifier in QIIME2 (v 2024.2), which is based on the SILVA *16S rRNA* gene database (v 138).

All data analyses were conducted on the Majorbio Cloud platform. The alpha diversity indices (e.g., Chao1 and Shannon) were calculated using mothur software (v 1.45.3). Then, the intergroup differences in alpha diversity were analyzed using the Wilcoxon rank-sum test; nonmetric multidimensional scaling (NMDS) based on Bray–Curtis distances was used to examine the similarity of microbial community structures among samples, combined with analysis of similarities (ANOSIM) to test for significant differences in community structure between groups; linear discriminant analysis effect size (LEfSe) (http://huttenhower.sph.harvard.edu/LEfSe, accessed on 10 August 2025) (LDA > 3, *p* < 0.05) was applied to identify bacterial taxa with significantly different abundances between groups at the phylum to genus levels. In addition, PICRUSt2 (v 2.4.1) predicted the functional abundance on the basis of *16S rRNA* gene sequencing data [32].

### 2.7. Statistical Analysis

Experimental data on intestinal digestive enzyme activity are presented as the mean ± standard error (mean ± SE). One-way analysis of variance (ANOVA) was performed using SPSS 28.0, with multiple comparisons conducted using the least significant difference (LSD) method. Differences were considered significant at *p* < 0.05.

## 3. Results

### 3.1. Feeding Organs and Intestinal Morphology

**Figure 2:**
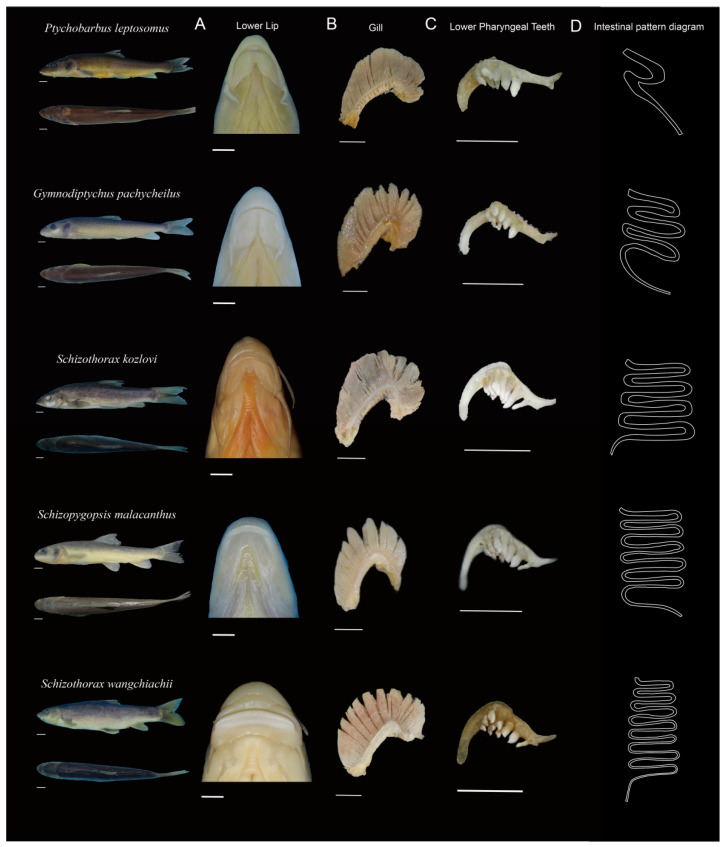
Feeding organs and overall morphology of five Schizothoracinae species. Scale bars in all panels represent 1 cm; (**A**), lower jaw; (**B**), gill rakers; (**C**), pharyngeal teeth; (**D**), schematic.

Significant morphological differentiation was observed in the main feeding organs and intestinal structures among the five Schizothoracinae species, with variations in the anterior margin of the lower jaw, pharyngeal tooth formula, number of outer gill rakers on the first gill arch, and number of intestinal flexures (Table 1). There were five differences here (Figure 2). (1) Anterior margin of the lower jaw: *Ptychobarbus leptosomus* (PL), *Gymnodiptychus pachycheilus* (GP), and *Schizothorax kozlovi* (SK) exhibited no keratin on the anterior margin of the lower jaw and had thick fleshy lips; *Schizopygopsis malacanthus* (SM) and *Schizothorax wangchiachii* (SW) had sharp keratinized anterior margins (Figure 2A). (2) Number of outer gill rakers on the first gill arch: PL had the fewest gill rakers; GP and SK had moderate numbers; and SM and SW had the highest counts (Figure 2B). (3) Pharyngeal tooth formula: PL, GP, and SM possessed two rows of pharyngeal teeth, whereas SK and SW had three rows. PL, GP, and SM each had 14 pharyngeal teeth, whereas SK and SW had more (20 teeth each) (Figure 2C). (4) Pharyngeal tooth morphology: all five species had pointed pharyngeal tooth apices. PL, GP, and SM presented narrow chewing surfaces, whereas SK and SW presented moderately wide chewing surfaces (Figure 2C). (5) Number of intestinal flexures: PL had the fewest flexures; GP and SK had moderate numbers; SM and SW had the most (D, Table S1).

### 3.2. Diet Composition

**Figure 3:**
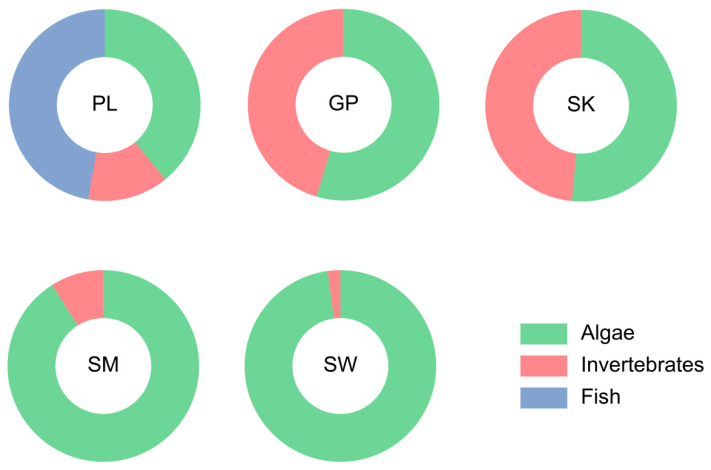
Diet compositions of five Schizothoracinae species. PL, *Ptychobarbus leptosomus*; GP, *Gymnodiptychus pachycheilus*; SK, *Schizothorax kozlovi*; SM, *Schizopygopsis malacanthus*; SW, *Schizothorax wangchiachii*.

On the basis of dietary composition (Figure 3), five species were classified into three trophic types: PL (omnivorous–carnivorous), with a high proportion of small fish in its diet; GP and SK (omnivorous), which primarily consume algae and invertebrates; and SM and SW (omnivorous–herbivorous), with algae as the main food source. Therefore, five species exhibit the distinct dietary compositions. Among them, the diet of *P. lelongatus* is particularly unique because it contains fish, making it a piscivorous species.

### 3.3. Intestinal Digestive Enzyme Activities

**Figure 4:**
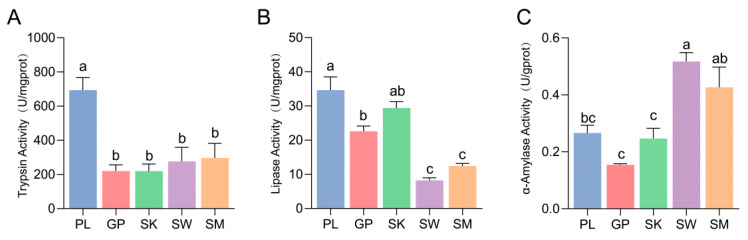
Intestinal digestive enzyme activities in five Schizothoracinae species. (**A**), Trypsin activity. (**B**), lipase activity. (**C**), α-amylase activity. PL, *Ptychobarbus leptosomus*; GP, *Gymnodiptychus pachycheilus*; SK, *Schizothorax kozlovi*; SM, *Schizopygopsis malacanthus*; SW, *Schizothorax wangchiachii.* The different lowercase letters in the bar chart indicatd significant differences.

The trypsin activity was significantly higher in PL than in the other four species (Figure 4A). The lipase activity was significantly lower in SM and SW than in PL, GP, and SK (*p* < 0.05) (Figure 4B). Conversely, the intestinal α-amylase activity was significantly lower in PL, GP, and SK than in SM and SW (*p* < 0.05) (Figure 4C).

### 3.4. Intestinal Microbial Diversity

After quality filtering, 4,351,166 sequences were obtained from 61 samples, including 33,113 amplicon sequence variants (ASVs), 54 phyla, 640 families, and 1770 genera. The samples were normalized to 29,523 sequences per sample to balance the sequencing depth.

#### 3.4.1. Intestinal Microbial Composition and Differential Analysis

**Figure 5:**
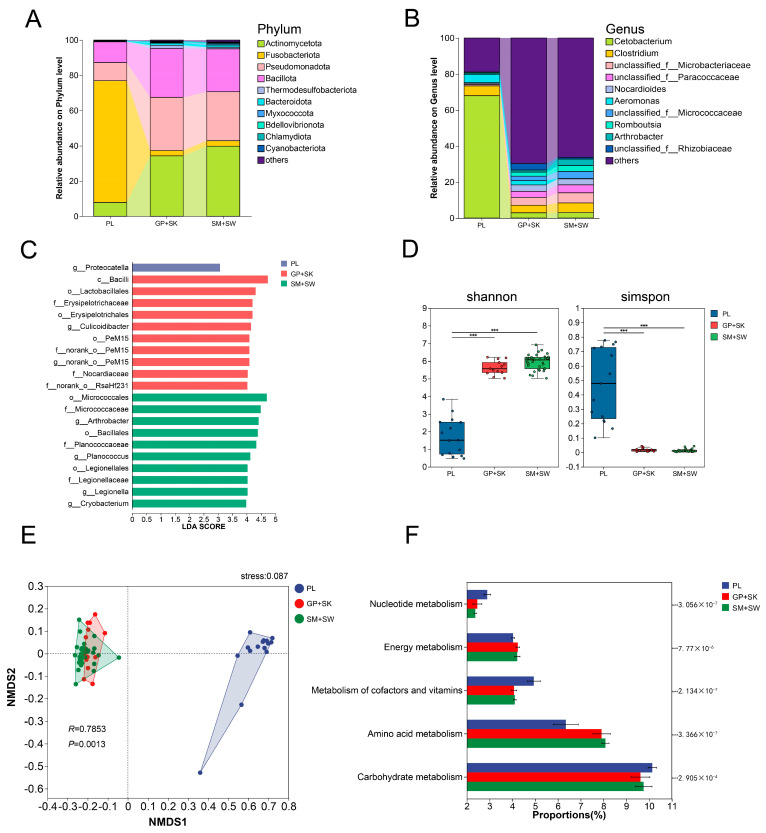
Analysis of intestinal microbial composition and diversity: (**A**), relative abundance of intestinal microbiota at the phylum level among different trophic groups; (B), relative abundance at the genus level; (**C**), LEfSe analysis of the intestinal microbiota; (**D**), α diversity of the intestinal microbiota; (**E**), NMDS analysis of the intestinal microbiota; (**F**), metabolic function prediction of the intestinal microbiota (based on KEGG level 2 pathways). PL, *P. leptosomus*; GP, *G. pachycheilus*; SK, *S. kozlovi;* SM, *S. malacanthus*; SW, *S. wangchiachii.* ***, means *p* < 0.001.

On the basis of the dietary results (Section 3.2), PL presented a distinct dietary composition relative to the other four species; in contrast, similar dietary compositions were observed between GP and SK, as well as between SM and SW. Thus, the five species were grouped into PL, GP + SK, and SM + SW for comparative analysis of intestinal microbial diversity. The *16S rRNA* gene sequencing revealed the composition of the intestinal microbiota in these groups. Five phyla with >1% average relative abundance were identified (Figure 5A): Actinobacteriota (PL, 5.09%; GP + SK, 38.15%; SM + SW, 39.80%), Fusobacteriota (PL, 73.67%; GP + SK, 2.23%; SM + SW, 3.09%), Pseudomonadota (PL, 9.34%; GP + SK, 28.56%; SM + SW, 27.78%), Bacillota (PL, 11.16%; GP + SK, 27.01%; SM + SW, 24.35%), and Bacteroidota (detected only in GP + SK, 1.44%, and SM + SW, 1.20%).

At the genus level, PL had five genera with >1% relative abundance: *Cetobacterium* (72.52%), *Clostridium* (5.60%), *Aeromonas* (4.95%), *Carnobacterium* (1.40%), and *Acinetobacter* (1.05%). GP + SK contained 15 such genera: *Clostridium* (4.61%), *Nocardioides* (3.73%), *Romboutsia* (2.63%), *Hyphomicrobium* (2.23%), *Cetobacterium* (2.22%), *Rhodococcus* (2.01%), *Culicoidibacter* (1.98%), *norank_o__PeM15* (1.89%), *Carnobacterium* (1.87%), *Oryzihumus* (1.49%), *Enterococcus* (1.48%), *norank_o__RsaHf231* (1.46%), *Arthrobacter* (1.27%), *Companilactobacillus* (1.04%), and *norank_o__Gaiellales* (1.04%). Finally, SM + SW had 14 genera meeting the abundance criterion: *Clostridium* (5.35%), *Arthrobacter* (3.67%), *Romboutsia* (3.30%), *Nocardioides* (3.28%), *Cetobacterium* (3.08%), *Planococcus* (1.90%), *Legionella* (1.67%), *Cryobacterium* (1.42%), *Hyphomicrobium* (1.38%), *norank_f__67-14* (1.36%), *Exiguobacterium* (1.29%), *norank_o__PeM15* (1.10%), *norank_o__Gaiellales* (1.07%), and *Oryzihumus* (1.01%) (Figure 5B).

The LEfSe revealed that *Proteocatella* and *Hafnia–Obesumbacterium* were significantly different in PL compared with those in GP + SK and SM + SW; *Culicoidibacter* and *norank_o__PeM15* were differentially abundant in GP + SK; and *Arthrobacter, Planococcus,* and *Legionella* were distinct in SM + SW (Figure 5C).

#### 3.4.2. α and β Diversity

The α diversity indices were calculated for the three groups (Figure 5D). In PL, the Shannon index was significantly lower and the Simpson index was significantly higher than those of GP + SK and SM + SW (Kruskal–Wallis H test, *p* < 0.01). No significant differences in the Shannon or Simpson indices were observed between the GP + SK and SM + SW groups (Kruskal–Wallis H test, *p* > 0.05).

ANOSIM and NMDS were performed to further assess intergroup microbial differences (Figure 5E). ANOSIM indicated R > 0 between PL and GP + SK, as well as between PL and SM + SW, suggesting greater intergroup than intragroup variation. NMDS analysis revealed significant separation of PL from GP + SK and SM + SW (stress = 0.087, *p* = 0.001), whereas the GP + SK and SM + SW samples overlapped extensively.

#### 3.4.3. Functional Prediction

PICRUSt2-based KEGG functional enrichment analysis revealed that intestinal microbial genes in all five species were enriched primarily in nutrient metabolism pathways, including carbohydrate metabolism, amino acid metabolism, cofactor and vitamin metabolism, and energy metabolism. However, the enrichment abundances varied significantly (Figure 5F). Specifically, compared with GP + SK and SM + SW, PL presented significantly increased enrichment in carbohydrate metabolism and cofactor/vitamin metabolism (Kruskal–Wallis H test, *p* < 0.05) but reduced enrichment in amino acid metabolism and energy metabolism (Kruskal–Wallis H test, *p* < 0.05).

## 4. Discussion

### 4.1. The Differences in Feeding Organs and Intestinal Morphology

Most fish species exhibit distinct morphological characteristics associated with their feeding habits, making it possible to infer their diet on the basis of feeding organ traits [33,34]. Studies have shown that Schizothoracinae fish with keratinized mandibular margins, primarily scrape epiphytic algae [35,36]. In contrast, species with fleshy lips typically feed on benthic invertebrates [37]. *P*. *kaznakovi* and *P*. *dipogon*, which possess fleshy mandibular lips, mainly consume benthic invertebrates [35,38]. The lower jaw morphology, shape of pharyngeal bones, number of pharyngeal teeth rows, and mouth position of fish were strongly related to the food types [7]. In the present study, the anterior margin of the lower jaw, pharyngeal tooth formula, number of outer gill rakers on the first gill arch, and number of intestinal flexures were varied among five Schizothoracinae species. In addition, PL, GP, and SK exhibited thick, fleshy mandibular lips and shorter relative intestinal lengths, whereas SM and SW had sharp keratinized mandibular margins and longer relative intestinal lengths. The fleshy lips of PL, GP, and SK likely facilitate feeding on benthic invertebrates, whereas the keratinized margins of SM and SW are adapted for scraping epiphytic algae. These morphological traits align with their observed food composition: PL primarily consumed small benthic fishes and invertebrates; GP and SK fed on benthic invertebrates and algae; and SM and SW mainly consumed algae. Relative intestinal length is a key morphological index associated with feeding habits [39,40]. Purely herbivorous or detritivorous fish often have intestinal lengths exceeding 3 times their body length (up to 15–20 times in some cases), whereas omnivorous or herbivorous-leaning omnivorous fish typically have intestinal lengths 1–3 times their body length. Carnivorous fish generally have relative intestinal lengths <1 [41]. In this study, PL, GP, and SK had relative intestinal lengths of 1–3, whereas SM and SW had lengths >3. A longer digestive tract enables prolonged food retention, increasing nutrient absorption efficiency [42]. The longer intestines of SM and SW may thus represent an adaptation to their higher intake of plant-based components.

### 4.2. Gut Digestive Enzyme Activity

Herbivorous fish exhibit high amylase activity to digest plant-based foods, whereas carnivorous fish rely on high protease activity to break down animal proteins [10]. Omnivorous fish display balanced enzyme activity to accommodate diverse food sources [43]. α-Amylase is an enzyme responsible for decomposing carbohydrates such as starch in plant-based diets. The activity of α-Amylase is significantly greater in herbivorous fish than in the omnivorous species [44,45,46]. In our study, the α-amylase activity levels in SM and SW were significantly greater than those in PL, GP, and SK (*p* < 0.05). This discrepancy can be attributed to the natural feeding preferences of SM and SW, which primarily consume algae—dietary items rich in carbohydrates. The elevated amylase activity observed in these species likely facilitates efficient decomposition of such carbohydrate-rich food sources, aligning with their herbivorous trophic niche. In contrast, the lower intestinal α-amylase activity levels in PL, GP, and SK are probably linked to their greater reliance on animal-based diets. As detailed in Section 3.2, PL primarily preys on *Triplophysa* species and invertebrates, which are food items with low carbohydrate content. The reduced demand for carbohydrate digestion under such dietary conditions may have driven the evolutionary adaptation of “low α-amylase activity” in these species over time.

Lipase activity is directly associated with the digestion of lipid-rich animal-derived diets [47]. Within high-elevation river ecosystems, animal-derived food items—such as small fishes, chironomid larvae, and caddisfly larvae—typically exhibit greater lipid content than plant-based resources, including algae [48]. Consistent with this, PL, GP, and SK presented significantly greater lipase activity than SM and SW did (*p* < 0.05), a pattern that corresponds to their increased consumption of lipid-rich animal prey.

Complementing these observations on lipase activity, trypsin—a key enzyme for protein digestion—also reflects dietary reliance on high-protein resources [49]. PL demonstrated significantly elevated trypsin activity relative to the other four species (*p* < 0.05), a finding that can be plausibly attributed to its greater intake of protein-rich dietary components such as *Triplophysa* spp. and aquatic insects. Such adaptive divergence in digestive enzyme activity may mitigate interspecific resource competition through the optimization of energy acquisition strategies.

### 4.3. Gut Microbial Diversity

Differences in food sources lead to variations in the gut microbial composition among fish with distinct feeding habits. For example, the abundance of *Fusobacteria* is positively correlated with the host’s ability to digest proteins and peptides [50], whereas *Actinobacteria* are more abundant in herbivorous fish, potentially assisting in plant polysaccharide degradation [51]. In this study, *Fusobacteria* (73.67%) dominated the gut microbiota of the PL group, which primarily consumed animal-based foods. In contrast, the GP + SK and SM + SW groups were dominated by *Actinobacteria* (38.15% and 39.80%, respectively), *Pseudomonadota* (28.56% and 27.78%), and *Bacillota* (27.01% and 24.35%). The high *Fusobacteria* abundance in PL may enhance protein digestion, whereas the high *Actinobacteria* abundance in GP + SK and SM + SW likely supports the degradation of plant-based food.

The gut microbiota plays a crucial role in nutrient absorption and metabolism in fish [52]. The most enriched metabolic pathways predicted in the gut microbiota of fish with different feeding habits are similar and primarily involve amino acid metabolism, carbohydrate metabolism, and coenzyme and vitamin metabolism [53]. In particular, a study of three cold-water fish species with distinct dietary preferences reached similar conclusions [12]. Furthermore, we found that the functional enrichment of the gut microbiota in the PL, GP + SK, and SM + SW groups was associated primarily with nutrient metabolism, including carbohydrate metabolism, amino acid metabolism, vitamin and cofactor metabolism, and energy metabolism, which is consistent with findings from previous investigations. There were differences in the enrichment levels of gut microbial genetic functions in carbohydrate and lipid metabolism pathways among herbivorous, omnivorous, and carnivorous fish [11]. Additionally, significant differences in the enrichment levels of nutrient-metabolism-related pathways in the gut microbiota have been observed between sympatric *Schizothorax* species with divergent feeding preferences [54]. Our results similarly reflect this pattern. Compared with the GP + SK and SM + SW groups, the PL group presented significantly increased enrichment in nucleic acid metabolism, vitamin and cofactor metabolism, and carbohydrate metabolism pathways but significantly reduced enrichment in energy metabolism and amino acid metabolism pathways. As previously noted, the diet of PL contains a greater proportion of animal-based food, which is not only rich in protein but also a substantial source of energy. In contrast, GP + SK and SM + SW consume primarily aquatic insects and algae, respectively. Given the low availability of food resources in plateau rivers, this may lead to insufficient amino acid and energy intake, prompting their gut microbiota to increase amino acid metabolism and energy metabolism to meet host nutritional demands.

In total, the differences in the relative abundances of amino acid metabolism, lipid metabolism, and energy metabolism pathways in the gut microbiota among *Schizothorax* species with distinct dietary habits are closely linked to nutrient metabolism, facilitating host adaptation to the food-scarce plateau hydrological environment. Owing to the limitations of *16S rRNA* gene sequencing technology, this study can only infer the functions of the gut microbiota. Future research should employ metagenomics to directly identify key genes and their host strains and further investigate the underlying mechanisms of their functional roles.

## 5. Conclusions

This study revealed significant differences in the morphology of major feeding and digestive organs, as well as diet composition, among sympatric Schizothoracinae fishes. These differences, coupled with adaptive changes in gut digestive enzymes and microbial diversity driven by food source variation, collectively promote nutritional niche differentiation. This differentiation facilitates the coexistence of Schizothoracinae fishes in high-elevation river ecosystems.

## Figures and Tables

**Table 1:** Comparison of the morphological characteristics of the main feeding and digestive organs of five Schizothoracinae species.

Species Name	*Ptychobarbus leptosomus* (*n* = 15)	*Gymnodiptychus pachycheilus* (*n* = 7)	*Schizothorax kozlovi* (*n* = 6)	*Schizopygopsis malacanthus* (*n* = 20)	*Schizothorax wangchiachii* (*n* = 13)
Mouth position	Subterminal	Subterminal	Subterminal	Subterminal	Subterminal
Anterior margin of lower jaw	No keratin, well-developed lips	No keratin, well-developed lips	No keratin, well-developed lips	Sharp keratin	Sharp keratin
Pharyngeal tooth formula	3.4/4.3	3.4/4.3	2.3.5/5.3.2	3.4/4.3	2.3.5/5.3.2
Pharyngeal tooth morphology	Pointed apex, narrow chewing surface	Pointed apex, narrow chewing surface	Pointed apex, moderate chewing surface	Pointed apex, narrow chewing surface	Pointed apex, moderate chewing surface
Number of outer gill rakers on first gill arch	11–15	16–18	13–17	17–21	20–22
Number of intestinal flexures	2	6	7	10	13
Body length range (cm)	28.5 ± 9.3	33.4 ± 8.2	27.9 ± 10.7	29.4 ± 7.6	29.3 ± 4.8
Relative gut length	1.41 ± 0.2	1.57 ± 0.1	2.69 ± 0.3	4.22 ± 0.3	5.14 ± 0.6

## Data Availability

The data presented in this study are available on request from the corresponding author without any restrictions.

## Supplementary Materials

The following supporting information can be downloaded at: https://www.mdpi.com/article/10.3390/ani15223242/s1; Table S1: Total length, body length, and weight of five species for counting intestinal pattern diagrams.
